# Lifetime‐Engineered Carbon Nanodots for Time Division Duplexing

**DOI:** 10.1002/advs.202003433

**Published:** 2021-02-01

**Authors:** Ya‐Chuan Liang, Kai‐Kai Liu, Xue‐Ying Wu, Qing Lou, Lai‐Zhi Sui, Lin Dong, Kai‐Jun Yuan, Chong‐Xin Shan

**Affiliations:** ^1^ Henan Key Laboratory of Diamond Optoelectronic Material and Devices School of Physics and Microelectronics Zhengzhou University Zhengzhou 450001 China; ^2^ State Key Laboratory of Molecular Reaction Dynamics Dalian Institute of Chemical Physics Chinese Academy of Sciences 457 Zhongshan Road Dalian 116023 China

**Keywords:** carbon nanodots, information encryption, optical dultiplexing, phosphorescence

## Abstract

Optical multiplexing attracts considerable attention in the field of information encryption, optical probe, and time‐resolved bioimaging. However, the optical multiplexing based on rare‐earth nanoparticles suffers from heavy metal elements and relatively short lifetimes; sophisticated facilities are thus needed. Herein, time division duplexing based on eco‐friendly carbon nanodots (CNDs) with manipulative luminescence lifetimes is demonstrated. In a single green color emission channel, the luminescence lifetimes of the CNDs can be manipulated from nanosecond level to second level by introducing water, while the lifetime of the CNDs confined by a silica shell stays. Time division duplexing based on the CNDs and CNDs@silica with distinct lifetimes is realized and spatio‐temporal overlapping information is thus resolved. High‐level information encryption using the time division duplexing technology is realized. This work may promise the potential applications of CNDs in multi‐lifetime channels biological imaging, high‐density information storage, and anti‐counterfeiting.

## Graphical abstract

Time division duplexing based on CNDs and CNDs@silica with distinct lifetimes is realized and spatio‐temporal overlapping information is thus resolved, which has been used for high‐level information encryption.

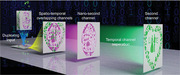

Time division multiplexing is that it uses time channel as a division object, and it is realized by allocating multiple channels with non‐overlapping time segments. Optical multiplexing has attracted considerable attention and plays an important role in the field of data storage, document encryption, anti‐counterfeiting, time‐resolved bioimaging, and more.^[^ ^1^, ^2^, ^3^, ^4^, ^5^, ^6^, ^7^ ^]^ To date, multiplexing using luminescence colors or intensity of fluorophores has been reported previously.^[^ ^8^, ^9^ ^]^ However, color or intensity based multiplexing is limited by the crowded spectral domain and background interference, leading to inevitable spectral overlap thus complicated optical imaging devices and tedious color compensations are required. Thus, luminescence lifetime as recognizable coding dimension has tremendous potential due to the capacious time domain.^[^ ^10^, ^11^ ^]^ Recently, tunable lifetime multiplexing using rare‐earth doped luminescent nanocrystals has been demonstrated by Lu et al.^[^ ^12^ ^]^ The new multiplexing concept by using decay lifetimes as temporal coding dimension is fascinating. However, the lifetimes are concentrated in the region of microsecond‐to‐millisecond, and their lifetimes are relatively short thus expensive time‐gated instrumentations and complicated data‐decoding processes are needed. Furthermore, the nanocrystals contain heavy metals, and controlling their lifetimes often requires complex preparation process, which limits their practical applications.^[^ ^13^, ^14^ ^]^ Therefore, developing eco‐friendly luminescent nanoparticles with easy to manipulate lifetime in a capacious time region is a key point for simplifying the coding and decoding process of multiplexing. It is an important ability to accurately control the luminescence lifetime for the enhancement of the optical multiplexing capability. Carbon nanodots (CNDs), as recently emerging luminescent nanoparticles, have been widely applied in light‐emitting diodes,^[^ ^15^, ^16^, ^17^, ^18^ ^]^ cell imaging,^[^ ^19^, ^20^, ^21^, ^22^ ^]^ information encryption,^[^ ^23^, ^24^, ^25^, ^26^, ^27^, ^28^, ^29^, ^30^, ^31^ ^]^ and biosensing^[^ ^32^, ^33^, ^34^, ^35^ ^]^ because of their excellent optical performance, environment‐friendliness, and high stability.^[^ ^36^, ^37^, ^38^ ^]^ Considering capacious luminescent lifetime domain and eco‐friendly performance of CNDs, they are ideal candidates for optical multiplexing. Yang et al. prepared blue emissive CNDs using citric acid and ethylenediamine as starting materials, and various patterns have been demonstrated by using prepared CNDs as fluorescence ink, but the lifetime of the CNDs is only in the range of nanosecond.^[^ ^39^ ^]^ Deng et al. reported phosphorescent CNDs via embedding CNDs in a polyvinyl alcohol (PVA) matrix for the first time and encrypted information has been realized by using the phosphorescent CNDs.^[^ ^40^ ^]^ Yu et al. demonstrated zeolite‐confined carbon dots with long lifetime, and security protection application has been achieved.^[^ ^41^ ^]^ CNDs with different lifetimes always are synthesized in different systems; the intensity and luminescence peaks are diverse, which are adverse to time division multiplexing of a single color channel. Furthermore, the encrypted information are easy to be cracked when UV light is off. In addition, straightforward lifetime‐engineering for CNDs remains a significant challenge, as it generally requires complex molecular design and elaborate excited‐state manipulation. It is still a huge challenge for CNDs to use time division mulplexing technology to resolve spatio‐temporal overlapping information.

In this work, time division duplexing based on the lifetime‐engineered CNDs (From nanosecond level to second level range) has been demonstrated; spatio‐temporal overlapping information is thus resolved. By adjusting triplet state of the CNDs and CNDs@silica using water, the lifetime of the CNDs is easy to be manipulated. The information cannot be resolved due to the spatio‐temporal overlapping channels before addition of water; while the temporal overlapping channels are separated by introducing water. The designed duplexing technology using lifetime‐engineered CNDs has been applied for advanced information (spatio‐temporal overlapping information) encryption. This work will expand their potential applications in the multi‐lifetime channels biological imaging, high‐density information storage, and anti‐counterfeiting.

The time division duplexing techology in this work is mainly based on the CNDs and CNDs@silica with controlled lifetime. In our previous work,^[^ ^20^ ^]^ CNDs with short fluorescence lifetime (Nanosecond level) and long phosphorescence lifetime (Second level) in solid state have been prepared. The fluorescence with short lifetime originates from the radiative recombination of singlet state, while the phosphorescence with long lifetime stems from the triplet radiative recombination. Generally, triplet excitons of the CNDs are easy to be deactivated in an aqueous solution due to the vibration of the bonds of the CNDs and the electron transition between CNDs and dissolved oxygen. It has been proved that confining the CNDs in silica to form CNDs@silica can hold their long lifetime in aqueous solution. The lifetime variation of the CNDs and CNDs@silica lays a solid foundation for time division multiplexing. The designed time division duplexing using CNDs and CNDs@silica is illustrated in **Figure** **1**a. The CNDs and CNDs@silica in the solid state show blue fluorescence and green phosphorescence, while only CNDs@silica can keep long lifetime phosphorescence in aqueous solution. The lifetimes of CNDs in powder and aqueous solution were 1.27 s and 1.98 ns, and the lifetimes of the CNDs@silica are 1.45 and 1.57 s, respectively, in solid state and aqueous solution. The lifetime‐resolved CNDs and CNDs@silica make it possible for time division duplexing. To realize duplexing, two channels marked with CNDs and CNDs@silica, respectively, are input in the same space, and the channels overlap in spatial‐temporal domain due to the similar lifetime of the CNDs and CNDs@silica in solid state. To decode the overlapping channels, water is introduced into this system, the lifetime of the CNDs decreases from second to nanosecond, while the lifetime of CNDs@silica can keep. In this case, channels are separated to nanosecond and second channel, which are easy to be detected by common camera, as shown in Figure 1b. The optimal preparation conditions of the CNDs@silica are investigated from two aspects (Tetraethyl orthosilicate (TEOS) concentration and preparation time), as shown in Figures S1 and S2, Supporting Information. The phosphorescence intensity of the CNDs@silica with different TEOS are shown in Figure S1a, Supporting Information. The phosphorescence intensity and lifetime of the CNDs@silica reaches a maximum when the TEOS volume was 0.5 mL. The phosphorescence intensity and lifetime of the CNDs@silica gradually increases and then almost keeps as the increase of the hydrolysis time and the optimal hydrolysis time is 8 h. (Figure S2, Supporting Information). As a result, the optimal synthesis process of the CNDs@silica is as follows: 1 mL CNDs aqueous solution and 0.5 mL TEOS were dispersed in 15 mL deionized water (DI) to form aqueous solution. Then, 0.5 mL ammonia was added into the above solution stirring for 8 h at room temperature. The possible chemical reactions process between TEOS and CNDs is discussed: The gelation process of silica can be described by two types of reactions, generally referred to as the hydrolysis and subsequent condensation reaction. In Figure S3, Supporting Information, TEOS can be hydrolyzed by water to form Intermediate Product 1 (IP1) (Reaction 1), and then the hydroxyl groups onto the surface of CNDs can react with TEOS according to reaction 2. Finally, the CNDs can be encapsulated in silica to form CNDs@silica. Fourier transform infrared (FTIR) spectra of the CNDs@silica with different TEOS hydrolysis times have been measured, as shown in Figure S4, Supporting Information. FTIR spectra reveal an absorption band at 3450 cm^−1^ assigned to the SiO‐H stretching vibration from the CNDs@silica. Upon incorporation of CNDs into the silica matrix, further characteristic vibrations appear for Si‐O‐Si (1050–1200 cm^−1^) and Si‐C (1500 cm^−1^), indicating that covalent bonds between the CNDs and external silica have formed. The covalent bonds between silica and the CNDs can efficiently limit molecular rotation and vibration. The morphology and structure of the CNDs@silica with different hydrolysis time were characterized by transmission electron microscope (TEM), as shown in Figure S5, Supporting Information. From the TEM images, one can see that the nanoparticles are gradually coated by amorphous silica shell with the increase of hydrolysis time. The CNDs display quasi‐spherical shape with diameter of about 4–5 nm and the lattice spacing is 0.21 nm, as shown in Figure S6, Supporting Information. As for the CNDs@silica, amorphous silica shell around the CNDs can be observed. The scanning electron microscope (SEM) image of the CNDs@silica has also been taken, as shown in Figure S7, Supporting Information. The CNDs@silica display quasi‐spherical shape.

**Figure 1:**
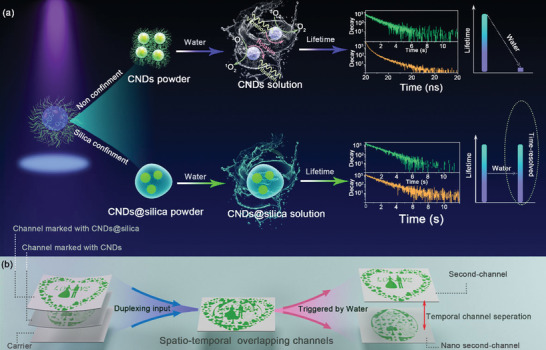
a) Schematic diagram and the corresponding luminescence lifetime of the CNDs and CNDs@silica in the solid state/aqueous solution. b) Schematic illustration of the time division duplexing based on the CNDs and CNDs@silica.

X‐ray photoelectron spectroscope (XPS) characterizations were carried out to investigate the elements and bonding state of the CNDs and CNDs@silica, as shown in Figure S8, Supporting Information. Correspondingly, full XPS spectra (Figure S8a, Supporting Information) reveal the existence of C, N, O, and P elements in the CNDs and a newly emerging Si peak can be observed in the XPS spectrum of CNDs@silica, indicating the presence of Si in the CNDs@silica sample. The C 1s spectra of the CNDs and CNDs@silica can be deconvoluted into two peaks at 284.6 and 286.3 eV, which are corresponding to C‐C and C‐O bonds,^[^ ^42^, ^43^, ^44^ ^]^ respectively. From N 1s spectra, two peaks at 399.4 and 401.3 eV are visible, which can be assigned to C‐N and N‐H^[^ ^45^ ^]^ (401.3 eV). The P‐O (530.6 eV) and P‐O (532.8 eV) bonds can be observed in the O 1s spectrum,^[^ ^26^ ^]^ and the Si 2p spectrum reveals the presence of Si‐O (104 eV) and C‐Si (104.8 eV). The P 2p spectrum contains two peaks centered at 133.1 eV and 134.9 eV for P‐O/P‐O, and P‐N bonds. Comparing with the high resolution XPS spectra of the CNDs (Figure S8a–f), the XPS spectra of the CNDs@silica prove the formation of Si‐O‐C and Si‐O‐Si bonds. Altogether, the XPS data provide clear evidence that the CNDs and silica have combined through covalent bonds. The controlled lifetime of the CNDs creates a lifetime dimension that can be used for time division duplexing, and the corresponding photophysical properties are investigated. The fluorescence (FL) excitation–emission contour plots of the CNDs powder are recorded, as shown in **Figure** **2**a. The CNDs powder with emission peak located at around 410 nm and the optimal excitation wavelength centered at about 365 nm. The normalized UV–vis absorption and excitation spectra of the CNDs powder are shown in Figure S9a, Supporting Information. A strong absorption peak at around 350 nm can be observed in UV–vis absorption spectra, which can be assigned to the n‐π* transition.^[^ ^46^, ^47^, ^48^ ^]^ Figure 2b shows time‐resolved emission spectra (TRES) versus times, revealing time‐independent fluorescence emission property. The lifetime of CNDs is in the range of nanosecond scale under aqueous solution ambient. The excitation‐phosphorescence emission contour plots of the CNDs powder are shown in Figure 2c. Phosphorescent emission with a peak at around 520 nm can be observed and the optimum excitation wavelength is about 350 nm and the quantum yield (QY) is 6.1%. The corresponding fluorescence excitation‐emission, time‐resolved emission, excitation‐phosphorescence emission contour plots of the CNDs@silica are shown in Figures 2d,e,f. UV–vis absorption and FL spectra were recorded in Figure S9b, Supporting Information, which are found to be similar to those of the CNDs powder, indicating that there is no change in aspect of photophysical properties after confining CNDs in silica nanospace. The phosphorescence QY of CNDs@silica increases to 12.6% after confining the CNDs into a silica nanospace. In order to test whether the CNDs and CNDs@silica can be used for time division duplexing, square patterns using CNDs and CNDs@silica as ink were printed on a filter paper. The printed patterns cannot be observed by the naked eye because the reflective UV–vis spectra of the filter paper and the CNDs‐printed areas are almost the same (Figure S10, Supporting Information) under sunlight. The patterns show blue and green emission in mode of UV on/off. The patterns printed with CNDs only show blue fluorescence after spraying water onto the surface of paper due to the phosphorescence quenching after meeting with water, while the patterns printed with CNDs@silica have no obvious change after water treatment; the corresponding images are shown in Figure 2g. The phosphorescence quenching of the CNDs indicates the lifetime of the CNDs decreases from second to nanosecond, which can be used for time division duplexing.

**Figure 2:**
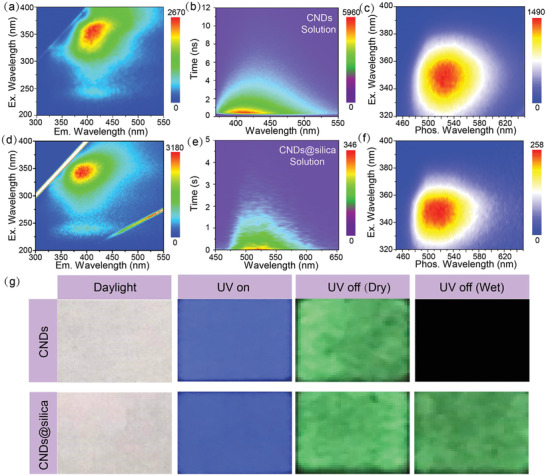
a) Fluorescence excitation–emission contour plots for the CNDs powder. b) Time‐resolved emission spectra of the CNDs powder. c) Phosphorescence excitation–emission contour plots for the CNDs powder. d) The excitation–emission contour plots for the CNDs@silica solution. e) Time‐resolved emission spectra of the CNDs@silica solution. f) Phosphorescence excitation–emission contour plots for the CNDs@silica. g) Fluorescence and phosphorescence images using CNDs and CNDs@silica as ink.

In order to investigate lifetime variation mechanism of the CNDs after meeting with water, the generation of singlet oxygen by CNDs and CNDs@silica was detected chemically using the singlet oxygen sensor green (SOSG) as a detector. The fluorescence of SOSG will be quenched due to the intramolecular electron transfer, while endoperoxide will format after reacting with singlet oxygen and the electron transfer is prohibited, thus green fluorescence of SOSG is then recovered. For the CNDs mixed with SOSG under illumination of UV light for 2 min, the SOSG fluorescence intensity exhibits increased enhancement with the concentration of CNDs from 10 to 60 mg mL^−1^ (**Figure** **3**a), indicating singlet oxygen can be generated through electron transfer between triplet excitons of the CNDs and triplet oxygen in aqueous solution. In contrast, the SOSG mixed with CNDs@silica shows low fluorescence intensity, suggesting that silica can block electron transfer between triplet excitons of dissolved triplet oxygen. To directly prove the evidence of singlet oxygen generation, the content of singlet oxygen was detected by electron paramagnetic resonance (EPR) spectra, as shown in Figure 3b. Singlet oxygen can be captured by singlet oxygen trap to generate radical, which can be detected by EPR spectrometer. The signal intensity of the CNDs aqueous solution is higher than that of the CNDs@silica aqueous solution, indicating singlet oxygen is generated through electron transfer between triplet excitons of the CNDs and triplet oxygen in aqueous solution. The corresponding schematic illustration for the electron transfer between the CNDs and oxygen are illustrated in Figure 3c. Ground state of oxygen is triplet state, and electron transfer is easy to occur between ground state oxygen and triplet state of the CNDs, leading to triplet oxygen transformation to singlet oxygen. In addition, the vibration of the CNDs will intensify in aqueous. Thus, dissolved oxygen and intensified vibration are the main reasons for phosphorescence quenching. Silica around the CNDs can isolate the CNDs with surrounding oxygen and limit the vibration of the CNDs, thus the emission lifetime of the CNDs is still in the range of second sacle in aqueous solution. The overall luminescence schematic diagrams of the CNDs and CNDs@silica under aqueous condition are illustrated in Figure 3d. The phosphorescence emission of the CNDs originates from radiative transitions of triplet excitons, and triplet excitons are easy to be deactived due to the long lifetime. In addition, in aqueous solution, molecule vibration of the CNDs will be intensified and electron transition between CNDs and dissolved oxygen will quench the phosphorescence of the CNDs.^[^ ^49^ ^]^ As a result, water can make temporal separation between the CNDs and CNDs@silica, which is favor of time division duplexing. The stability of the security inks is a key issue that determines the performance of the time division dulplexing, thus the stability of the CNDs and CNDs@silica is accessed. The fluorescence and phosphorescence intensity can keep under illumination of UV light (0.15 mW cm^−2^) for 8 h, as shown in Figure 3e and Figure S11, Supporting Information, indicating their excellent photostability. In addition, they can be repeatedly activated with no obvious loss of luminescence intensity (Figure 3f and Figure S12, Supporting Information), demonstrating their excellent signal consistency. The fluorescence and phosphorescence stability of CNDs@silica in aqueous solution under different pH values has been measured, as shown in Figure S13, Supporting Information. The fluorescence intensity of the CNDs@silica shows a little change with the pH decrease from 10 to 1, and the phosphorescence intensity decreases in acidic and alkaline environment. Thus, the optimal pH value for the CNDs@silica in aqueous is 7. To quantitatively evaluate the stability of security inks based on the CNDs and CNDs@silica under dry and wet conditions, the CNDs and CNDs@silica solution used as ink were simultaneously printed onto a fluorescer‐free paper, and five repeated water‐spraying treatment cycles were studied. As shown in Figure S14, Supporting Information, CNDs@silica inks can maintain phosphorescence emission and the emission of the CNDs show very good reversibility with desiccating and wetting treatments. In Fiugre 3g, there is no phosphorescence intensity change of the CNDs@silica under nitrogen and air atmosphere, which indicates their immunity to oxygen. Such an oxygen‐immunity phosphorescence characteristic is distinct from the reported phosphorescence that can be quenched by oxygen in aqueous solution; this may be attributed to the silica isolation effect. The images printed on filter paper can be observed after 3 months under ambient conditions (Figure S15, Supporting Information). Moreover, no remarkable changes in the legibility of images were observed after heat treatment at 40 °C for 24 h (Figure S16, Supporting Information). The phosphorescence intensity of the CNDs@silica printed onto paper almost remained constant under illumination of UV irradiation for 30 min, as shown in Figure S17, Supporting Information. The above results indicate that the CNDs@silica have high stability, suggesting it is suitable to application of stable time division duplexing.

**Figure 3:**
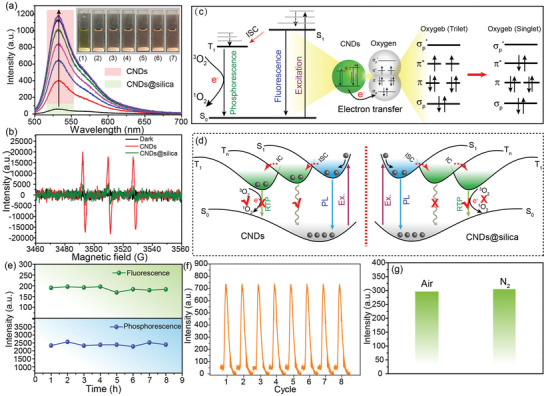
a) The fluorescence spectra of SOSG solution treated with CNDs (10, 20, 30, 40, 50, and 60 mg mL^−1^) and CNDs@silica (100 mg mL^−1^). b) EPR spectra of CNDs and CNDs@silica aqueous solution upon irradiation. c) The triplet excitons transition model between the CNDs and dissolved oxygen in aqueous solution. d) Overall luminescence schematic diagrams of the CNDs and CNDs@silica under aqueous condition. e) The fluorescence and phosphorescence intensity of the CNDs@silica under illumination of UV for 8 h. f) The phosphorescence intensity of the CNDs@silica solution as a function of the cycle number of UV activation. g) The phosphorescence intensity of the CNDs@silica solution in air and nitrogen ambient.

For realizing time division duplexing, channel‐1 and channel‐2 information in the same space should have different time scales. Thus, channel‐1 information was printed with CNDs as ink, channel‐2 information was printed with CNDs@silica as ink. The corresponding schematic illustration and time‐resolved emission spectra are shown in **Figure** **4**. Under dry condition, the channel‐1 pattern shows blue fluorescence and green phosphorescence, and second‐scale luminescence can be observed from time‐resolved spectra, as shown in Figure 4a. The long lifetime phosphorescence of the CNDs within paper is due to immobilization effect of fiber. Under water vapor treatment, the phosphorescence of the CNDs within paper will be quenched and only fluorescence can be detected (In the right of Figure 4b), which is due to the intensified molecule vibration and triplet state electron tranfer between CNDs and dissolved oxygen. The TRES show that the emission lifetime of the CNDs decreases to 6 ns. For the channel‐2 pattern printed using CNDs@silica as ink, it still shows blue fluorescence and green phosphorescence even spraying water onto surface of pattern; the TRES can also confirms that the emission lifetime of channel‐2 pattern is in the range of second scale, as shown in Figure 4c. It is suggested that the prepared CNDs and CNDs@silica are suitable for time division duplexing.

**Figure 4:**
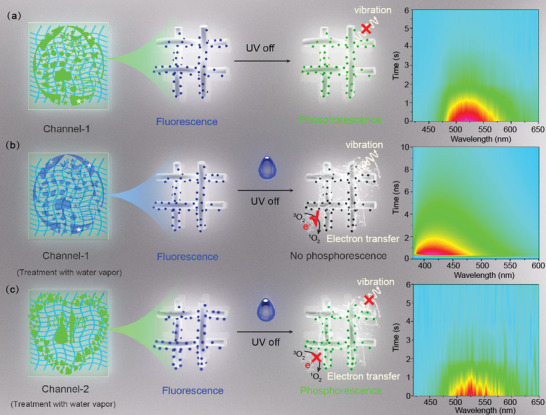
a) Schematic illustration of Channel‐1 information printed onto paper using CNDs as ink and the corresponding time‐resolved spectra under dry condition. b) Schematic illustration of Channel‐1 information printed onto paper using CNDs as ink and the corresponding time‐resolved emission spectra after water vapor treatment. c) Schematic illustration of Channel‐2 information printed onto paper using CNDs@silica as ink and the corresponding time‐resolved emission spectra after water vapor treatment.

Time division duplexing technology for advanced information security based on water‐sensitive and water‐immune emission lifetime of the CNDs and CNDs@silica has been demonstrated. The CNDs and CNDs@silica aqueous solution were injected into an inkjet printer (Canon MG2580S printer) as ink; the printing process is shown in Figure S18, Supporting Information. The channel‐1 pattern and channel‐2 pattern using CNDs and CNDs@silica solution as ink were simultaneously printed onto a fluorescer‐free paper, thus duplexing information was input in same space. Channel‐1 and channel‐2 patterns show similar emission lifetime; the two channels overlap in spatial‐temporal domain, which cannot be cracked. For realizing time division duplexing, water was introduced into this system. The overlapped channels are separated to nanosecond and second channels after treatment with water vapor; the channel‐1 and channel‐2 information are thus read out, all of the processes are illustrated in **Figure** **5**a. An advanced information encryption has been demonstrated based on the above time division duplexing technology. Two channels with different patterns (Figure 5b) or text (Figure 5c) as specific information are printed into a paper, and they cannot be resolved in UV illumination (I) and UV off (II) modes due to the similar emission color and lifetimes. In order to separate the overlapped channels, they must be treated with water vapor. The emission lifetime of channel‐1 pattern or text decreases to nanosecond, while that of channel‐2 pattern or text can still be in the second scale, thus channel‐1 and channel‐2 information is separated in temporal domain. The corresponding separated patterns or texts (Nanosecond and second channel) are shown in III and IV of Figure 5b,c. Notice that the channel‐2 information can be obtained by a common camera, and the channel‐1 information is achieved through a subtract program between overlapped channel‐1 and channel‐2. Time division duplexing based on emission lifetime‐controlled CNDs and CNDs@silica has been realized, which can be used for high security level information encryption and storage. The influences of different humidity on the legibility of recorded information were investigated. As shown in Figure S19, Supporting Information, the legible characters of Christmas tree (using CNDs@silica as ink) and panda (using CNDs as ink) remained almost unchanged for 1 h under 50 % humidity. Although the characters of a Christmas tree and panda became a little faded when the security paper was stored under 80 % humidity for 1 h, it could also be clearly discerned by the naked eye. The above result indicates that the time division duplexing applications using CNDs and CNDs@silica as inks can be retained for a long time at relatively high humidity.

**Figure 5:**
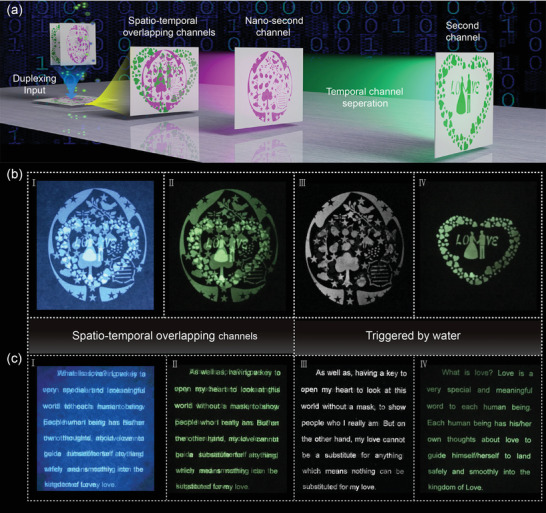
a) Schematic illustration of the time division duplexing based on the CNDs and CNDs@silica. b) The spatio‐temporal overlapping patterns (I‐II), and temporal separated patterns (III‐IV) based on time division duplexing technology. c) The spatio‐temporal overlapping text (I‐II), and temporal separated text (III‐IV) based on time division duplexing technology.

In summary, time division duplexing based on phosphorescence lifetime controlled CNDs and CNDs@silica has been demonstrated for the first time, which is independent of luminescence color and intensity. Water plays a key role for regulating the luminescence lifetime of the CNDs through quenching the triplet excitons. Spatio‐temporal overlapping channel‐1 and channel‐2 information have nine orders of magnitude (From nanosecond to second) in time domain; thus it can be resolved by a common camera. High‐level encryption or high‐density information storage has been demonstrated based on proposed time division duplexing. The result reported in this paper may open a door for the potential application of CNDs in optical multiplexing technology.

## Experimental Section

##### Characterization

TEM images were recorded on JSM‐6700F TEM at 200 kV. PL and phosphorescence spectra were performed, obtained on Hitachi F‐7000 spectrophotometer. The lifetimes of the CNDs were measured by time correlated single photon counting on a FLS980 spectrometer with excitation of 350 nm at room temperature. Fourier transform infrared (FTIR) spectra were obtained on a Nicolet 6700 spectrometer (Thermo Fisher Scientific, USA). The X‐ray photoelectron spectra (XPS) were obtained using PERKIN ELMZR PHI 3056.

##### Materials

The precursors used in this study included ethylenediamine (EDA, purity >99%), phosphoric acid (purity >95%), tetraethoxysilane (purity >99.9%), ammonia, and deionized water. All of the chemicals were purchased from Macklin Chemistry Co. Ltd (Shanghai, China). Note that all the chemicals used in this work were analytical grade without further purification.

##### Synthesis of CNDs

1.0 mL of EDA was first added in a beaker with 15 mL deionized water, and then 2 mL of phosphoric acid was slowly added into the EDA aqueous solution with stirring for 30 min. The formed transparent solution was then heated in a microwave oven (750 W) for 130 s. 20 mL deionized water was added into above samples after the samples cooled down to room temperature and then the yellow solution was formed. The sediment was removed through centrifuge and the supernatant was filtered through 0.22 µm membrane and then neutralized by sodium carbonate. The supernatant was collected and subjected to dialysis (MWCO: 500 Da) for a week. Finally, pale yellow solid CND powder was obtained by freeze‐drying.

##### Synthesis of CNDs@silica

1 mL CNDs aqueous solution and 0.5 mL tetraethoxysilane (TEOS) were dispersed in 15 mL deionized water to form aqueous solution. Then, 1 mL ammonia liquor was injected into the above solution stirring at room temperature for 8 h. Finally, transparent s CNDs@silica solution was obtained.

##### Time Division Duplexing for Advanced Encryption

Two different patterns were printed onto a paper with CNDs and CNDs@silica as ink through layer‐by‐layer printing method. The filter paper was used as substrate and the channel‐1 information was constructed by painting the aqueous solution of CNDs on the filter paper. Lastly, channel‐2 was created by printing the CNDs@silica ink onto the top of channel‐1.

##### Photoluminescence (PL) Quantum Yield (QY) Measurement

The PL QY of the samples was measured by a FLS980 spectrometer with a calibrated integrating sphere in. In principle, the PL QY can be expressed by the following Equation:

(1) $$\eta =\frac{\varepsilon }{\alpha }=\frac{\int_{390}^{750}L_{\mathrm{emission}}}{\int E_{\mathrm{reference}}-\int E_{\mathrm{sample}}}=\frac{\mathrm{number}\;\mathrm{of}\;\mathrm{emitted}\;\mathrm{photons}}{\mathrm{number}\;\mathrm{of}\;\mathrm{absorbed}\;\mathrm{photons}}$$

where *η* is the PL QY, *ε* is the emitted photons of the sample that were collected by the integrating sphere in the range of 390–770 nm, and *α* is the photons absorbed by the sample. *L*_emission_ is the net luminescence of the sample. *E*_reference_ is the absorption spectrum of the reference in the sphere. *E*_sample_ is the absorption spectrum of the sample and reference. The calculated PL QY of the CNDs and CNDs@silica in the range of 390–770 nm is about 13.3% and 27.3% based on above equation. The proportion of phosphorescence QY is about 46% in the total PL QY, thus the phosphorescence QY of the solid‐state CNDs and CNDs@silica are about 6.1% and 12.6%, respectively

## Conflict of Interest

The authors declare no conflict of interest.

## Supporting information

Supporting Information
