# Supplementary material for: Lifetime‐Engineered Carbon Nanodots for Time Division Duplexing
Source: Adv Sci (Weinh). 2021 Feb 1;8(6):2003433. doi: 10.1002/advs.202003433 (PMC7967062; doi:10.1002/advs.202003433)
Supplement: Supplementary file 1 — Supporting Information [file ADVS-8-2003433-s001.pdf]

© 2020 Wiley-VCH GmbH

ADVANCED SCIENCE Open Access

Supporting Information

for *Adv. Sci.*, DOI: 10.1002/advs.202003433

Lifetime-Engineered Carbon Nanodots for Time Division Duplexing

Ya-Chuan Liang, Kai-Kai Liu, Xue-Ying Wu, Qing Lou, Lai-Zhi Sui*, Lin Dong, Kai-Jun Yuan, Chong-Xin Shan**

*To whom any correspondence should be addressed, Email: liukaikai@zzu.edu.cn, lzsui@dicp.ac.cn, cxshan@zzu.edu.cn

DOI: 10.1002/ ((please add manuscript number))
Article type: **Communication**

Lifetime-Engineered Carbon Nanodots for Time Division Duplexing

Ya-Chuan Liang, Kai-Kai Liu[*]*, Xue-Ying Wu, Qing Lou, Lai-Zhi Sui*[*]*, Lin Dong, Kai-Jun Yuan, Chong-Xin Shan*[*]

Mr. Y. Liang, Dr. K. Liu, Mrs. X. Wu, Dr. Q. Lou, Prof. L. Dong, Prof. C. Shan
Henan Key Laboratory of Diamond Optoelectronic Material and Devices, School of Physics and Microelectronics, Zhengzhou University, Zhengzhou 450001, China.
E-mail: liukaikai@zzu.edu.cn. cxshan@zzu.edu.cn.
Dr. L. Sui, Prof. K. Yuan
State Key Laboratory of Molecular Reaction Dynamics, Dalian Institute of Chemical Physics, Chinese Academy of Sciences, 457 Zhongshan Road, Dalian, 116023, China.
E-mail: lzsui@dicp.ac.cn

[*]To whom any correspondence should be addressed, Email: liukaikai@zzu.edu.cn, lzsui@dicp.ac.cn, cxshan@zzu.edu.cn

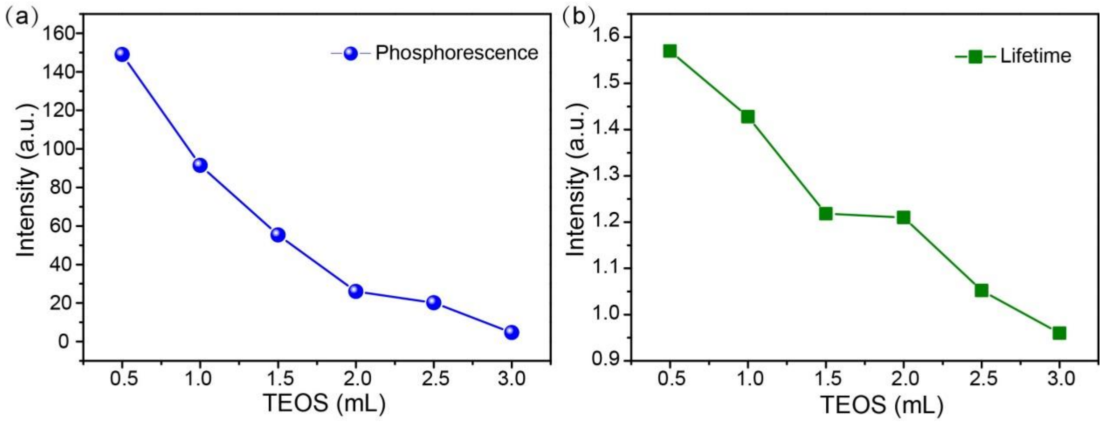

Figure S1. (a) The phosphorescence intensity and lifetime (b) of the CNDs@silica with different TEOS volumes

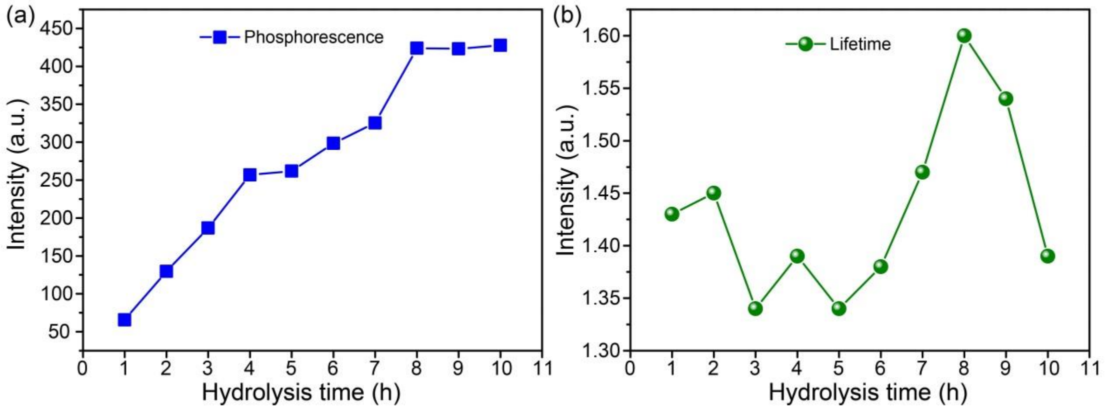

Figure S2. (a) The phosphorescence intensity and lifetime (b) of the CNDs@silica with different hydrolysis times.

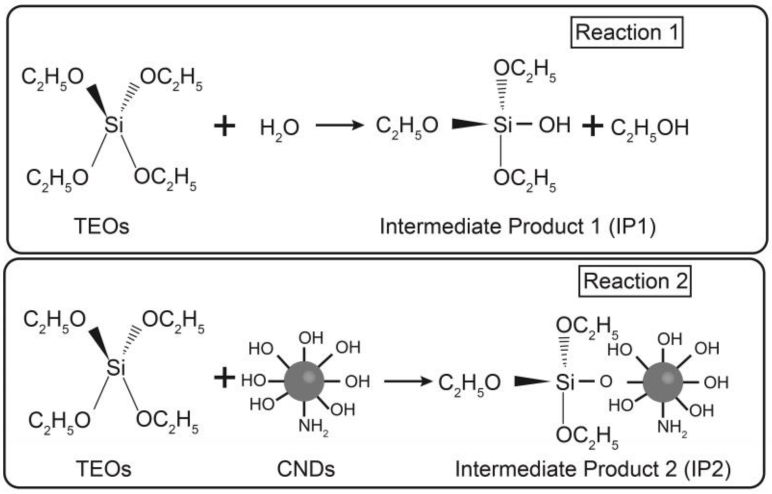

Figure S3. The silica encapsulation mechanism of the CNDs@silica.

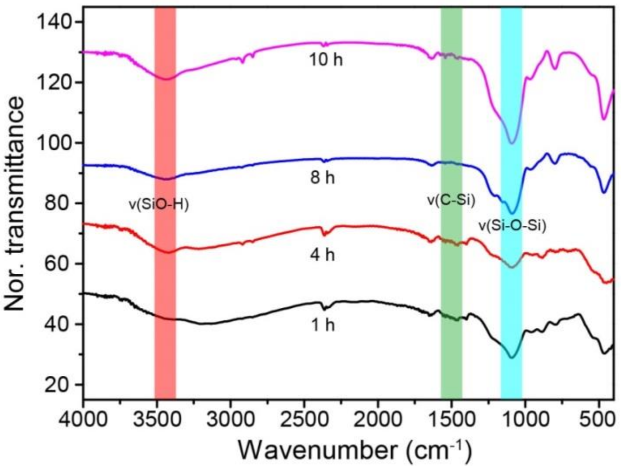

Figure S4. The FTIR spectra of CNDs@silica with different TEOS hydrolysis times.

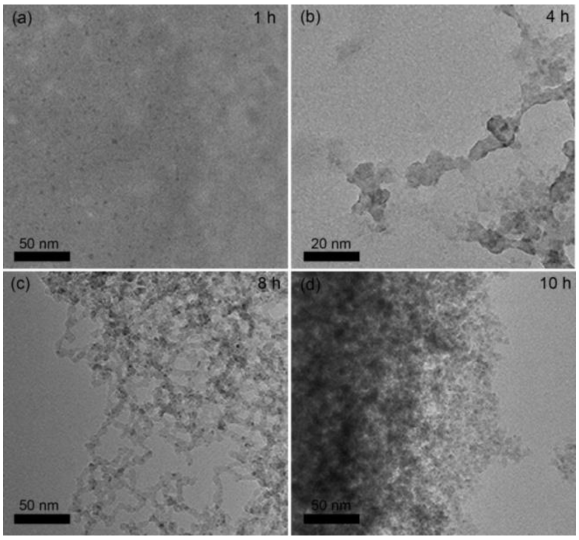

Figure S5. The TEM images of the CNDs@silica with different TEOS hydrolysis times.

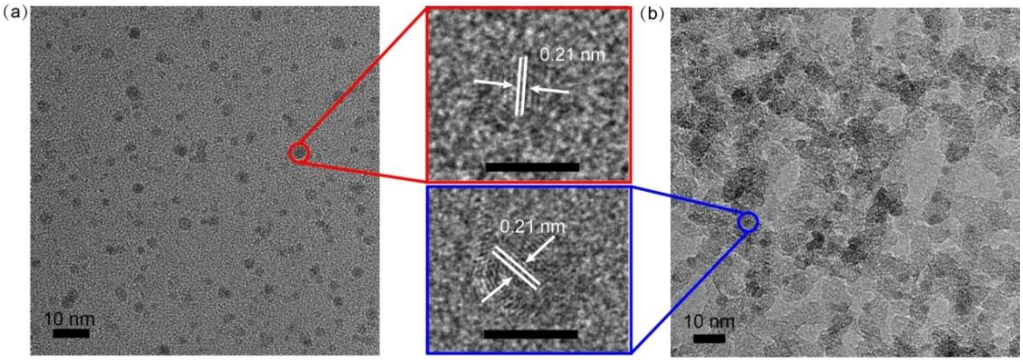

Figure S6. TEM image of the CNDs (a) and CNDs@silica (b).

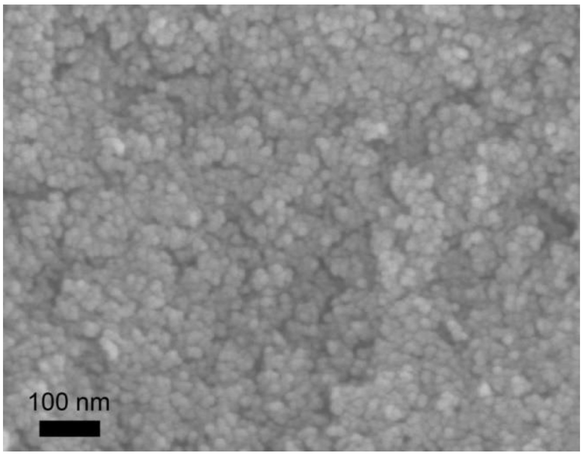

Figure S7. The SEM image of the CNDs@silica.

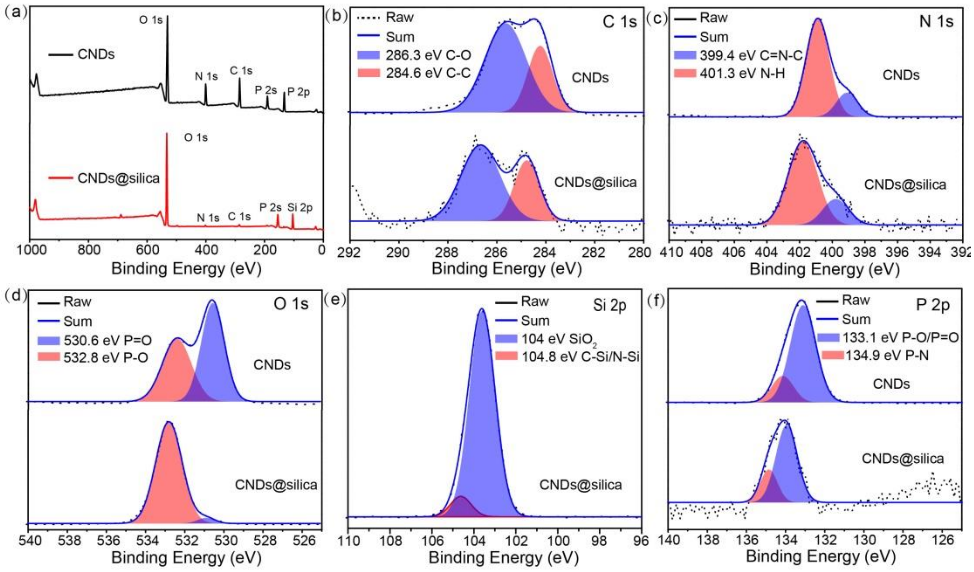

Figure S8. (a) The XPS spectra of the CNDs and CNDs@silica. (b-f) High-resolution C 1s (b), N 1s (c), O 1s (d), Si 2p (e) and P 2p (f) spectra of CNDs and CNDs@silica, respectively.

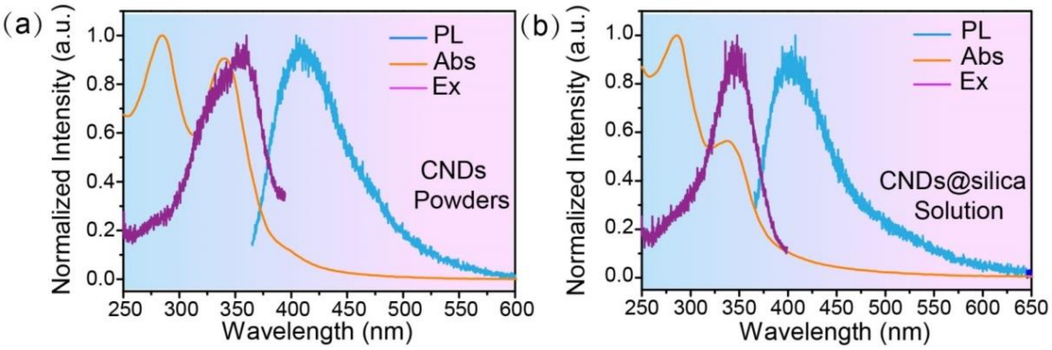

Figure S9. (a) The normalized UV-vis absorption, PL emission and excitation spectra of the CNDs powder. (b) The normalized UV-vis absorption, FL emission and excitation spectra of the CNDs@silica solution.

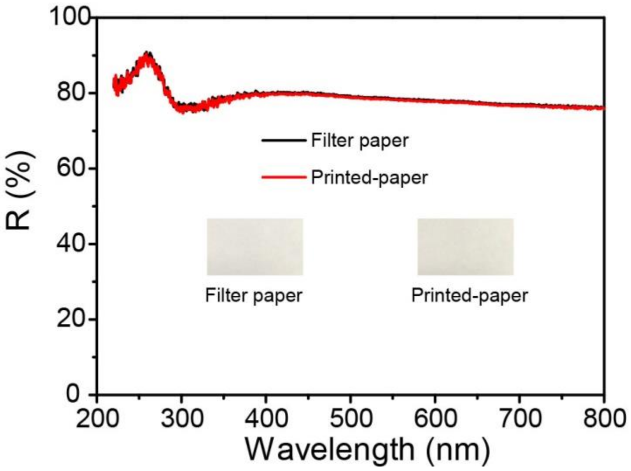

Figure S10. The reflective UV-vis spectra of the filter paper and the CNDs-printed areas.

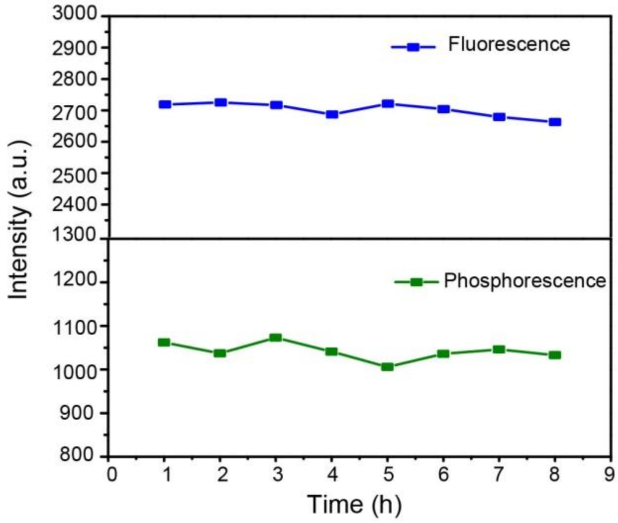

Figure S11. The fluorescence and phosphorescence intensity of the CNDs under continuous UV illumination for 8 h.

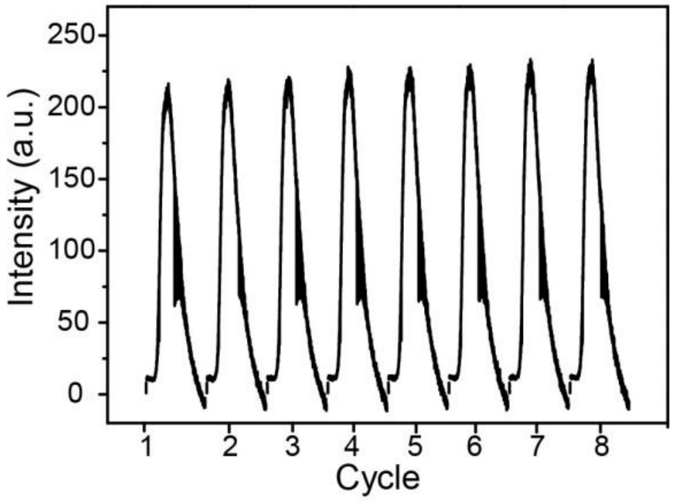

Figure S12. The phosphorescence intensity of the CNDs solution as a function of the cycle

number of illumination.

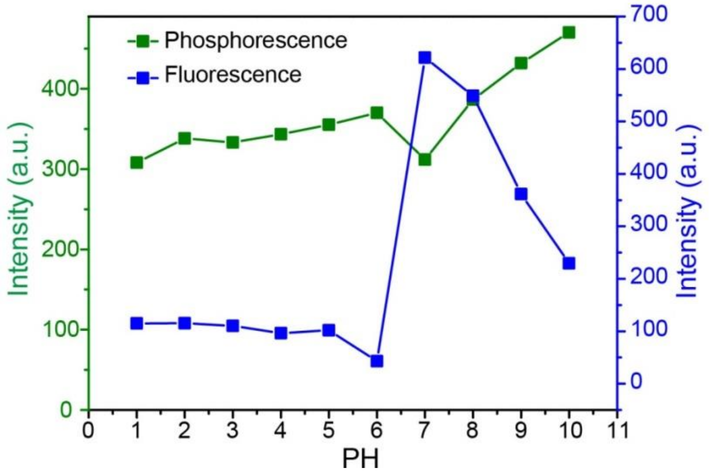

Figure S13. The fluorescence and phosphorescence intensity of the CNDs@silica in aqueous solution under different pH values.

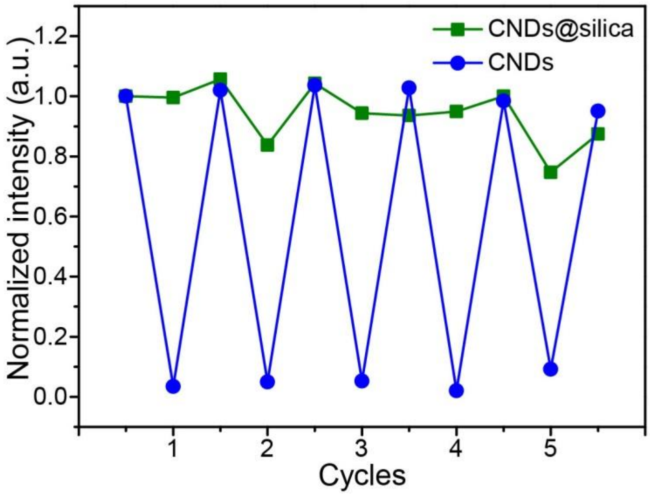

Figure S14. Phosphorescence intensity of printed patterns using the CNDs and CNDs@silica as ink under dry and wet conditions for five cycles.

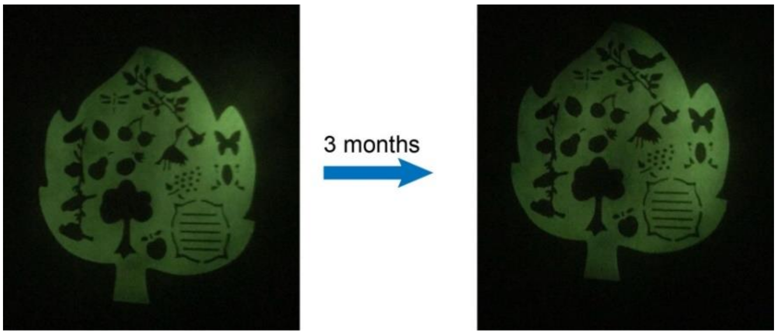

Figure S15. The images printed on filter paper after 3 months under ambient conditions.

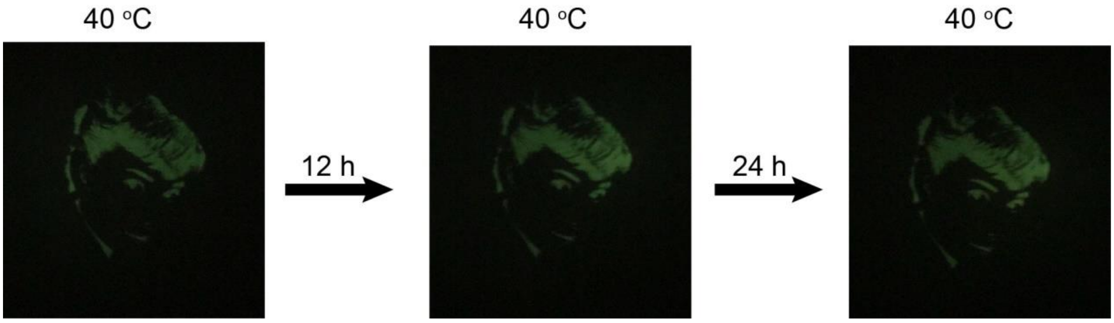

Figure S16. The images printed on filter paper after storing at 40 ^{o}C in oven for 24 h.

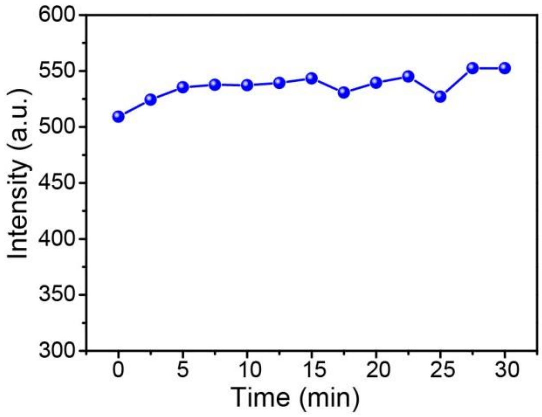

Figure S17. The phosphorescence intensity of the CNDs@silica at 520 nm in filter paper under continuous UV irradiation at 365 nm for 30 minutes.

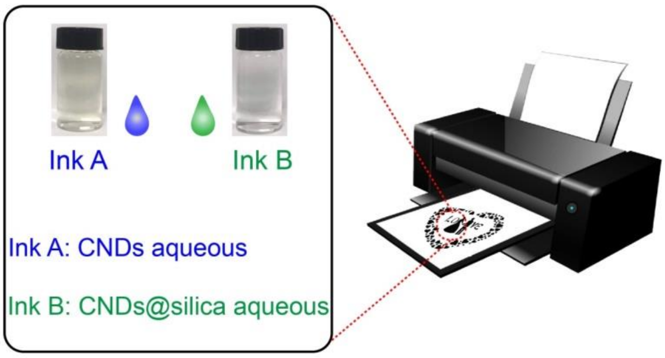

Figure S18. Schematic illustrations of printing process using a customized tricolor inkjet.

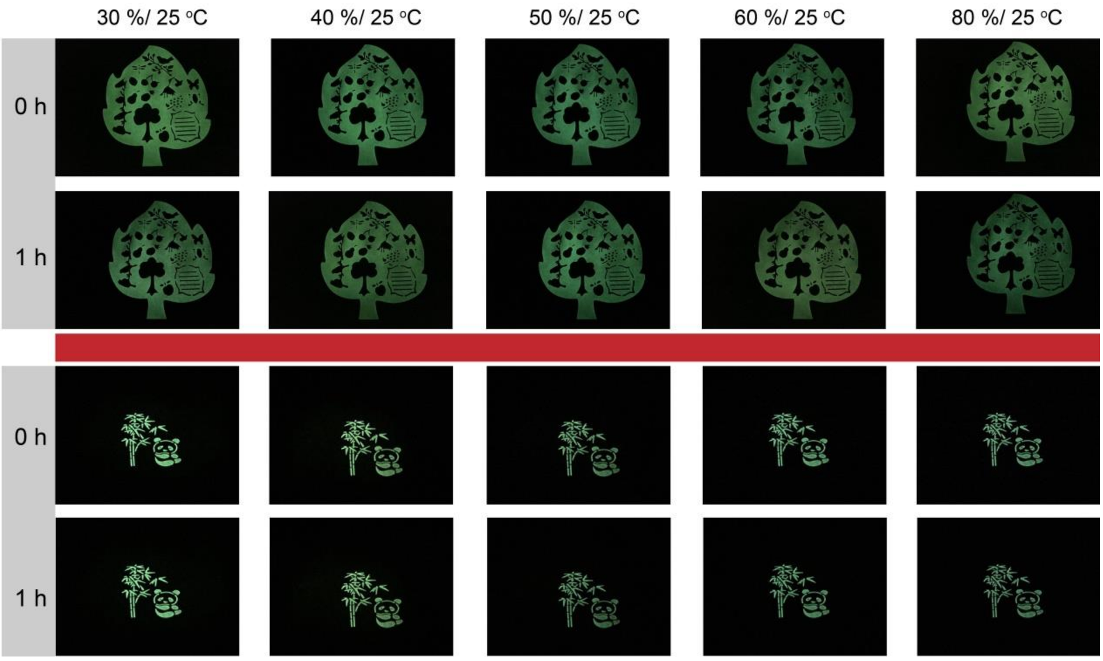

Figure S19. The recorded information using CNDs and CNDs@silica as ink at various humidity of 30%, 40%, 50%, 60%, and 80% for 0 h and 1 h.
